# Selection Signatures in Italian Goat Populations Sharing the “*facciuto*” Phenotype

**DOI:** 10.3390/genes16040390

**Published:** 2025-03-28

**Authors:** Simona Tarricone, Nikola Schlosserová, Silvia Bruno, Maria Teresa Sardina, Vincenzo Landi, Francesco Giannico, Maria Antonietta Colonna, Francesca Maria Sarti, Emiliano Lasagna, Simone Ceccobelli, Salvatore Mastrangelo, Paola Crepaldi, Fabio Pilla, Elena Ciani, Marco Ragni

**Affiliations:** 1 Dipartimento di Scienze Del Suolo, Della Pianta E Degli Alimenti, Università degli Studi di Bari “Aldo Moro”, 70125 Bari, Italy; 2 Dipartimento di Bioscienze, Biotecnologie e Ambiente, Università degli Studi di Bari “Aldo Moro”, 70125 Bari, Italy; 3 Dipartimento di Scienze Agrarie, Forestali, Alimentari e Ambientali, Università degli Studi della Basilicata, 85100 Potenza, Italy; 4 Dipartimento di Scienze Agrarie, Alimentari e Forestali, Università degli Studi di Palermo, 90128 Palermo, Italy; 5 Dipartimento di Medicina Veterinaria, Università degli Studi di Bari “Aldo Moro”, 70010 Valenzano, Italy; 6 Dipartimento di Scienze Agrarie, Alimentari e Ambientali, Università degli Studi di Perugia, 06121 Perugia, Italy; 7 Dipartimento Scienze Agrarie, Alimentari e Ambientali, Università Politecnica delle Marche, 60131 Ancona, Italy; 8 Dipartimento di Scienze Agrarie e Ambientali-Produzione, Territorio, Agroenergia, Università degli Studi di Milano, 20133 Milano, Italy; 9 Dipartimento di Agricoltura, Ambiente e Alimenti, Università degli Studi del Molise, 86100 Campobasso, Italy

**Keywords:** goats, “*facciuto*” phenotype, SNPs, selection signatures, pigmentation, coat color

## Abstract

**Background:** The presence of light-pigmented facial stripes, parallel on both sides of the cranial region, is a widespread characteristic in various goat breeds of European origin and beyond. In Italy, this phenotype is relatively common from the north to the south of the peninsula. The availability of genotypic data at single-nucleotide polymorphism (SNP) loci for breeds and populations characterized by such a pigmentation pattern enabled us to study the genomic regions potentially correlated with this phenotype, for simplicity referred to as “*facciuto*”. **Methods**: We adopted an F_ST_-outlier approach to detect signals of differential selection in 18 pairwise comparisons, each involving 6 genetic goat types with the “*facciuto*” phenotype (Facciuta Lucana, Facciuta della Valnerina, Valfortorina, Teramana, Capestrina, and Roccaverano) contrasted with each of 3 “*non-facciuto*” goat breeds selected as reference populations (Red Mediterranean, light brown; Saanen, white; Malagueña, mahogany solid). **Results**: The analysis of the region ±200 kbps upstream and downstream of the two significant signals on chromosome 13 and 15 allowed us to identify, among the annotated genes, *ASIP*, *AHCY*, *ITCH*, *DYNLRB1*, *MAP1LC3A*, *PIGU*, *LOC102177263*, and *DTX4*, whose functions could be related to several mechanisms underlying the phenotype under investigation. **Conclusions:** This study confirmed the fundamental role of *ASIP* in pigmentation, although additional pathways may concurrently contribute to the determinism of the considered “*facciuto*” phenotype in Italian goats.

## 1. Introduction

The diversity and genetic mechanisms underlying coat coloration across different mammalian groups are relevant for the investigation and understanding of mammalian adaptive evolution [1,2]. Variations in coat color among mammals play vital functions in predation, mate selection, camouflage, social communication, recognition, and protection from UV radiation [3]. As an example, dark skin pigmentation with a light-colored coat have helped tropical cattle, in tropical climates, with the adaptability to solar radiation [4]. In mammals, the coat pattern is determined by quantity and distribution in the body of two types of melanin, pheomelanin (red/yellow pigments) and eumelanin (black/brown pigments) [5], which are also considered to be important antioxidants and photo-protective agents [6,7]. Pigmentation is a complex cellular process involving the differentiation and maturation of melanocytes, the genesis and maturation of melanosome structures, and the production and within- and across-cell transport of melanin. At every stage of pigmentation, crucial functional genes are involved, contributing to the formation of diverse coat colors through articulated regulatory networks [8]. Among the many genes impacting pigmentation and pigment patterning, some have been thoroughly investigated, primarily focusing on the melanin synthesis pathway, such as α-melanocyte-stimulating hormone (*α-MSH*) [9,10], agouti signaling protein (*ASIP*) [11], melanocortin 1 receptor (*MC1R*) [12], tyrosine protein related 1 (*TRP1*) [13], dopachrome tautomerase (*DCT*) [14], receptor tyrosine kinase (*KIT*) [15], and microphtalmia-associated transcription factor (*MITF*) [16]. The production of eumelanin by melanocytes relies on the presence of α-MSH. Melanocytes have surface receptors that bind α-MSH. When bound, α-MSH initiates a signaling cascade that activates adelylate cyclase, ultimately leading to eumelanin production. Without this activation, melanocytes produce pheomelanin by default. Thus, the balance between eumelanogenesis and pheomelanogenesis is finely regulated by the activity of α-MSH and its receptors [17]. ASIP is encoded by the agouti gene, one of the key loci regulating hair color in mammals [18]. It regulates the production of pheomelanin by inhibiting the binding of α-MSH to MC1R, and consequently, it blocks the production of the essential enzymes needed for eumelanin synthesis [19]. Moreover, along the MC1R signaling pathway, the required regulation of tyrosinase (*TYR*), tyrosine protein related 1 (*TRP1*), and dopachrome tautomerase (*DCT*) are initiated for eumelanin production [20]. Among melanin-related genes, *KIT*, a gene involved in melanoblast development, migration, and survival [21], is pivotal in the melanogenesis signaling pathway, and mutations or deletion of *KIT* can cause different hair and skin colors in mammals [22]. Finally, *MITF* is a transcription factor acting as a master regulator of melanogenesis. Indeed, its activation stimulates the transcription of several downstream pigmentation genes, such as *TRP1* and *DCT* [23]. These genes regulate key steps in melanogenesis, determining melanin production, pigment patterns, and distribution within organisms [24,25,26]. However, pigmentation involves many other genes impacting the different stages beyond melanin synthesis, including intra- and inter-cellular pigment transport, cellular interactions, and environmental responses. Many of these genes remain poorly studied or even unknown [27,28].

Human-driven domestication and selection have resulted in a wide range of goat breeds characterized by distinct traits [21], including a wide range of pigmentation phenotypes [5,29]. Identifying the genetic loci responsible for coat color variation in goats has been an active area of research interest [11,29,30,31,32]. These phenotypes include goats with (i) solid colors like black (e.g., Hainan black goats, Bengal goat), red (e.g., Kalahari), white (e.g., Chongming white goat, Angora goat, Saanen), and mahogany (e.g., Malagueña); (ii) diluted solid colors like gray (e.g., Jining gray goats) and light red (e.g., Red Sokoto); (iii) two solid color patterns (e.g., Valais blackneck goat, Coppernecked goat, Boer goat); and (iv) specific markings (e.g., black mask, Swiss markings, Bezoar) or white spotting (e.g., Barbari goats). Understanding the genetic bases of these phenotypic variations requires examining changes at the DNA sequence level. As an example, in goats, a genome-wide association study (GWAS), based on single-nucleotide polymorphism (SNP) data, revealed an association between brown coat color and a chromosomal region where *TRP1* is located [33]. Similarly, in 228 Markhoz goats, GWAS revealed significant associations between different coat color and chromosomal regions: notably, black and brown coat colors were linked to chromosome 13, where the *ASIP*, *ITCH*, *AHCY*, and *RALY* genes are located, while white coat color was associated with chromosome 6, which contains the *KIT* and *PDGFRA* genes [34]. In addition to sequence variations such as SNPs or small indels, prior studies have demonstrated that duplication or deletion of larger DNA segments (i.e., copy number variations, CNVs) can impact genomic regions associated with phenotypic traits in livestock, including pigmentation. As an example, CNVs at a goat’s *ASIP* locus may lead to changes in the expression level or activity of the agouti protein, resulting in modifications to distribution and intensity of coat pigmentation-mutated animals [30]. Henkel et al. [21] located two CNVs in the 3′-flanking region of *KIT*, which they associated with the white coat color phenotype in Pak Angora goats, as well as with the white spotted phenotype in Barbari goats. On the other hand, the locus causing the white spotting phenotype in South African Boer goats was mapped, through a GWAS approach, to chromosome 17, where a subsequent analysis revealed a 1 Mb CNV withholding five genes, including *EDNRA* [35]. Notably, concerning the “Swiss marking” phenotype in goats, which is characterized by primarily black or brown coat color, white facial markings on the muzzle, and a white underbelly and legs, a possible role on this trait by *ASIP* has been suggested by using a genome-wide association study (GWAS) [36] and an ROH (runs of homozygosity) approach [37]. Phenotypes showing a patterning highly similar to the “Swiss marking” are present in various goat breeds of non-Swiss origin, including a number of Italian breeds (available online at: https://www.agraria.org/ovini.htm, accessed on 11 February 2025), where this trait is generally indicated with the term “*facciuto*”, also known as “badgerface”, based on the nomenclature by Sponenberg et al. (1998) [38]. Pictures and a short description about the six “*facciuto*” breeds included in this study are presented in Supplementary Table S1. These are, from the north to south of Italy, (i) Roccaverano, (ii) Facciuta della Valnerina, (iii) Capestrina, (iv) Teramana, (v) Valfortorina, and (vi) Facciuta lucana. All of them are local breeds, characterized by relatively low population sizes. Four of the six breeds (i.e., Roccaverano, Capestrina, Valfortorina, and Teramana) are included in the “*Anagrafe nazionale della biodiversità di interesse agricolo e alimentare*” (National Register of Biodiversity of Agricultural and Food Interest), published by the Italian Ministry of Agriculture, Food Sovereignty and Forestry (MASAF) through Ministerial Decree (M.D.) No. 156997 of 15 March 2023. This register is a tool listing all the animal and plant genetic resources subjected to risk of extinction or genetic erosion. The above-mentioned breeds, as well as the Facciuta della Valnerina, are also included in the corresponding registers managed at the regional level. Finally, the Roccaverano, Capestrina, Valfortorina, Teramana, and Facciuta lucana breeds are also included in the “*Disciplinare del libro genealogico e del registro anagrafico della specie caprina*” (Regulations for the Genealogical Book and the Anagraphic Register of Goat Species), approved through M.D. No. 9319 of 23 April 2010. This tool, managed by the “*Associazione Nazionale della Pastorizia*” (National Association of Pastoralism; AssoNaPa), is fundamental for both the genetic improvement of Italian caprine breeds (through the genealogical book) and the implementation of genetic conservation programs (through the anagraphic register). Concerning the Facciuta della Valnerina, it has been recently recognized as a breed in the latest Goat Central Technical Commission of the AssoNaPa.

Specifically, the Roccaverano breed is native to the Piemonte region (Northern Italy). Its coat, generally long-haired, is of variable color: brown, black or white, solid or piebald. White coloration of the distal parts of the limbs is common, and two white streaks running from the eyebrow region to the lips are also common (R.A.R.E. association, available online at: https://www.associazionerare.it/, accessed on 11 February 2025). This breed is classified as “endangered” in the Domestic Animal Diversity Information System (DAD-IS), a FAO-developed database providing breed-related information for analyzing the diversity of livestock breeds, including their conservation status. The Facciuta della Valnerina is widespread in the Apennine area between the Marche and Umbria regions (Central Italy). It is characterized by a long black coat, light ends of the limbs and the distinctive feature of the two light stripes on the head. To the best of our knowledge, the current size of this breed is around 200–300 heads, of which a portion (N = 73) was subjected to a morphological characterization in 2013 [39]. For this breed, a detailed DAD-IS local risk status is not available as the breed is not included in the database. Capestrina is reared in the mountain area in the southern part of the Lazio region (Central Italy). This breed is characterized by a dark coat color (black or brown) and by two white supraorbital lines. It is classified as “endangered” in the DAD-IS. The Teramana goat breed is raised in the Abruzzo region (Central Italy) and takes its name from the province of Teramo, where the greatest concentration of these animals can be found. These are goats with dark coat (mainly black or dark brown) with the possibility of white streaks on the head [40]. According to the DAD-IS, its risk status is listed as “critical”. The Valfortorina breed is raised in the province of Benevento (Campania region, Southern Italy). The coat is white and tawny in color, and the head has the typical “*facciuto*” phenotype. It survives in very low numbers, and its conservation status is listed as “critical” in the DAD-IS. Facciuta lucana is a local goat breed from the Basilicata region (Southern Italy). It has a long black coat with reddish reflections, light-colored extremities of the limbs (white or light beige) and the presence of two light stripes on the muzzle [41]. In recent years, through the VAL.BI.OVI.CAP. research project, funded and supported by the Basilicata Region (PSR Basilicata 2014–2020), aiming at the recovery and enhancement of the autochthonous sheep and goat populations/breeds, it was possible to evaluate the numerical consistency of this breed, which currently contains about 200 subjects, present in the Basilicata region and on a single farm in the Apulia region (Laterza, Taranto). To date, a detailed DAD-IS local risk status for this breed is not available.

In addition to goats, the presence of light-pigmented facial stripes, parallel on both sides of the cranial region, is attested in other mammalian species such as horses [42], rabbits [43], dogs [44], and cattle [45], making the investigation of the molecular basis of this phenotype in goats particularly interesting, as it may represent a model to stimulate further comparative studies across species.

The goal of this study was to identify genes potentially under differential selection related to the “*facciuto*” phenotype in Italian goat breeds by adopting a multi-cohort F_ST_-outlier approach. Through this study, we aim to corroborate and, possibly, extend the current knowledge about the genetic mechanisms underlying the investigated phenotype.

## 2. Materials and Methods

### 2.1. Samples and Genotypic Data

A total of 263 animals, belonging to 9 breeds, were genotyped using the Illumina 52K Goat BeadChip (Table 1). Out of them, six breeds displayed the “*facciuto*” phenotype (Facciuta lucana, Facciuta della Valnerina, Valfortorina, Teramana, Capestrina, and Roccaverano; Supplementary Table S1), while the other three were characterized by a “*non-facciuto*” light brown (Red Mediterranean), white (Saanen), and mahogany (Malagueña) coat color phenotypes (see Supplementary Table S2 for more details).

The animals with the “*facciuto*” phenotype included in this study belong to six local breeds, characteristic of different regions of the Italian peninsula. Since in small local breeds the phenomenon of phenotypic variability is quite common [46], the animals had been carefully selected by expert evaluators for the presence of the phenotype under investigation (i.e., the two white stripes on the muzzle). Genotypic data for the Facciuta della Valnerina, Teramana, Capestrina, Roccaverano, Red Mediterranean, and Saanen breeds were obtained from Cortellari et al. (2021) [47]. Genotypic data for the Malagueña breed were obtained from the AdaptMap project [48]. Genotypes for Facciuta Lucana and Valfortorina were specifically generated as part of this study. For the latter, blood samples were collected in EDTA K3-coated vacuum tubes during routine veterinary procedures (art. 1, comma 5 of the Directive 2010-63-EU). The genomic DNA from whole blood was extracted using the FlexiGene DNA kit (Qiagen, Hilden, Germany), according to the manufacturer’s instructions. The DNA concentration and purity was assessed with the NanoDrop™ 2000c Spectrophotometer (Thermo Fisher Scientific, Waltham, MA, USA). Samples were genotyped using the Illumina 52K Goat BeadChip at the University of Palermo, Italy. Before downstream analyses, the following quality control criteria were applied to the raw genotype dataset using PLINK software v. 1.9 [49] in order to both increase the robustness of the results and minimize the risk of false positives: (i) loci with call rate ≤ 90% (*--geno* 0.1), (ii) loci with minor allele frequency ≤ 0.05 (*--maf* 0.05), (iii) individuals with genotyping rate ≤ 90% (*--mind* 0.1), and (iv) non-autosomal loci were removed.

### 2.2. Detection of F_ST_-Outlier Markers

We employed the F_ST_-outlier approach, as implemented in *BayeScan* v2.1 (available at https://cmpg.unibe.ch/software/BayeScan/, accessed on 11 February 2025) [50], to identify markers putatively under differential selection in “*facciuto*” vs. “*non-facciuto*” goat breeds. Notably, we conducted 6 pairwise comparisons for each of the “*non-facciuto*” breeds, for a total of 18 pair-wise comparisons (Table 1), by adopting the software’s default criteria (i.e., *-n 5000 -thin 10 -nbp 20 -pilot 5000 -burn 50,000 -pr_odds 10*). Loci with q-values < 0.05 in ≥ 50% of the contrasts (3/6) were retained as putative under differential selection, with the q-value being the minimum FDR at which each locus may become significant. To further reduce the risk of false positive signals, we arbitrarily decided to focus, for the downstream gene annotation analysis, only on those loci that were observed as putative under differential selection in at least two out of the three “*non-facciuto*” scenarios. For each SNP meeting these criteria, we extended our search both upstream and downstream within 200 kb intervals. Moreover, to additionally test the robustness of the observed results obtained as described above, *BayeScan* analyses were repeated by contrasting the six “*facciuto*” breeds merged into a single meta-population (“FAC”) with the three “*non-facciuto*” reference breeds and by applying the following cut-off criteria: loci with q-values < 0.05 and observed as putative under differential selection in at least two out of three “*non-facciuto*” scenarios. Finally, we estimated for the significant SNPs the minor allele frequency within the nine considered breeds using the PLINK command *--freq --within*.

### 2.3. Gene Content of Genomic Regions Identified as Under Selection

Gene content in genomic regions putatively under selection was defined using *Genome data Viewer* for *Capra hircus* (available online: https://www.ncbi.nlm.nih.gov/gdv/browser/genome/?id=GCF_001704415.2, accessed on 11 February 2025),) by using the *ARS1.2* as a reference genome. We then conducted a comprehensive review of the existing literature and public databases to explore the biological role and phenotypic effects associated with each annotated gene.

## 3. Results and Discussion

### 3.1. SNP Loci Under Differential Selection

After the quality control, the final working dataset included 49,009 SNP loci and 263 animals. The best candidate loci were identified through a multi-cohort F_ST_-outlier approach by performing a total of 18 pairwise comparisons among the 9 breeds included in the dataset (Table 1 and Supplementary Table S3). After applying the strict criteria described in the Materials and Methods section (Supplementary Table S4), two genomic loci were identified as putative under differential selection, on chromosome 13 and 15, respectively (Table 2). The above signals were also confirmed ad significant when repeating the BayeScan analysis contrasting the six “*facciuto*” breeds, merged into a single population, with the three “*non-facciuto*” comparison breeds (Supplementary Table S5). Minor allele frequencies for the two SNP loci within the 9 considered breeds are presented in Supplementary Table S6.

### 3.2. Best Candidate Regions and Putatively Selected Genes

The analysis of the arbitrarily defined intervals ±200 kb upstream and downstream of each significant SNP locus showed the presence of genes putatively under selection for the investigated phenotype (Table 2). The region on chromosome 13 included *ASIP*, *AHCY*, *ITCH*, *DYNLRB1*, *MAP1LC3A*, and *PIGU* as known genes, while the region on chromosome 15 included several uncharacterized loci, *LOC102177263*, annotated as mRNA macrophage-expressed gene 1 protein (*MPEG1*, *alias* Perforin-2), and the *DTX4* gene. Interestingly, all the annotated genes on chromosomes 13 and 15 had known literature evidence supporting their involvement in melanogenesis and/or in other coat color-related mechanisms (Supplementary Table S7). This evidence supports the robustness of our findings and points to the fact that the weak remaining background variability in general coat color in the considered breeds does not pose a problem, due to our multi-cohort approach focusing on signals shared across multiple breeds, similarly to what we did in previous published studies investigating coat color phenotypes in cattle [51,52,53]. However, we cannot exclude the possibility that the detected significant signals could also be related to traits other than the considered coat color specific patterning (“*facciuto*”). For instance, we noticed that, based on the observed within-breed minor allele frequency patterns (Supplementary Table S6), as well as on the results of the BayeScan analysis performed contrasting the six “*facciuto*” breeds, merged into a single meta-population, with the three “*non-facciuto*” comparison breeds (Supplementary Table S5), the SNP rs268286785 on chromosome 13 seems to better capture the genetic differentiation between our “*facciuto*” breeds and the Saanen and Malagueña reference breeds, while the SNP rs268264603 on chromosome 15 may better seize the genetic differentiation between the “*facciuto*” breeds and the Red Mediterranean (as well as the Malagueña). Considering that in the candidate region on chromosome 13 genes related to cranial development are mapped together with genes known for their involvement in protein turnover and pigmentation (see the text below), and that cranial appendage features exist that differentiate Saanen and Malagueña vs. Red Mediterranean (e.g., ear morphology), a possible interconnection between the two pieces of evidence cannot be disregarded.

The *ASIP* (agouti signaling protein) gene is known to be involved in the regulation of melanogenesis in several livestock species since it may act locally as an extracellular color modifier, thus influencing the distribution of the pheomelanin and eumelanin pigments on the body [5,54]. It is well known that melanogenesis is regulated by the binding of α-MSH or ASIP to MC1R. Notably, the link of ASIP to MC1R precludes α-MSH-initiated signaling, blocking the production of c-AMP, thus leading to a downregulation of the synthesis of dark eumelanins in favor of the synthesis of lighter pheomelanins [55]. Several *ASIP* variants cause a wide variety of coat color patterning in domestic animals. For instance, *ASIP* loss-of-function variants are responsible for recessive black coat color in dogs [56], horses [57], and rabbits [58], while *ASIP* gain-of-function variants lead to red phenotype in dogs [59]. Moreover, it was suggested [21] that the presence of CNVs in the ASIP gene or in its proximity could be involved in the determinism of the analogousness of the “*facciuto*” pattern in Swiss goat breeds (“Swiss marking”) by altering the mRNA expression levels of the *ASIP* gene, as well as of different coat color phenotypes in several livestock species, such as cattle [54], sheep [3], and buffaloes [60].

Although not directly related to pigmentation, the *AHCY* (adenosylhomocysteinase) gene is associated with vitiligo [61], an autoimmune disease resulting in depigmentation due to the loss of melanocytes from the epidermis, and it is involved in phenomena of anomalous embryonic development at the cranial level. Similarly, *ITCH* (itchy E3 ubiquitin protein ligase) is involved in facial dysmorphic conditions [62].

*DYNLRB1* (dynein light chain roadblock type 1) acts as one of several non-catalytic accessory components of the cytoplasmic dynein 1 complex that are thought to be involved in linking dynein to cargos and to adapter proteins that regulate dynein function. Cytoplasmic dynein 1 acts as a motor for the intracellular retrograde motility of vesicles and organelles along microtubules [63]. Notably, vesicle trafficking to the target organelles is controlled by two classes of microtubule-associated motor proteins, i.e., kinesins and cytoplasmic dyneins. Both classes of proteins have well-established roles in retrograde and in anterograde transport of melanosomes [64,65]. Interestingly, it has been demonstrated that retrograde trafficking mechanisms of melanosomal cargoes to the Golgi are involved in Hermansky–Pudlak syndrome [66,67], a human disorder characterized by oculocutaneous albinism (i.e., a condition affecting the production of melanin).

*MAP1LC3A* (*alias LC3*)*,* encoding microtubule-associated proteins, is a ubiquitin-like modifier playing a role in the formation of autophagosomes [68,69], double-membrane cytosolic vesicles involved in the mechanism of autophagy responsible for protein turnover.

The *PIGU* (phosphatidylinositol glycan anchor biosynthesis class U) gene is a component of the glycosylphosphatidylinositol (GPI) transamidase complex [70]. GPI-anchored proteins are critical for embryogenesis, neurogenesis, and cell signaling [71], and GPI absence is responsible for defective intracellular transport of melanosomal proteins from the Golgi complex to melanosomes [72]. It was demonstrated [73] that autophagy competes with GPI-anchor synthesis, highlighting the possible interplay between these pathways. In addition, the PIGU protein has been found to be related to human melanoma risk [74,75] and to hair color in the human European population [76].

*LOC102177263* (alias perforin-2) is involved in vitiligo. Notably, melanocyte-specific CD8+ T-cells are increased in the blood of patients with vitiligo compared to healthy controls [77,78,79], resulting in the killing of melanocytes in vitro via Fas-Fas ligand (FasL) signaling, or through the release of cytotoxic granules such as perforins [7,73].

Interestingly, both *ITCH* on chromosome 13 and *DTX4* (Deltex E3 ubiquitin ligase 4) on chromosome 15 encode E3 ubiquitin ligases, playing a role in the process of protein turnover through the ubiquitin–proteasome–autophagolysosomal system. The latter involves also the ubiquitin-like modifier *MAP1LC3A* gene. In addition, since the *DYNLRB1* gene is involved in the retrograde trafficking of melanosomes, it may play a role in the turnover of misfolded melanin pigments within melanosomes.

Taken together, this evidence supports the hypothesis that, as previously suggested in cattle [51], in goat species the absence or reduced pigmentation at the level of the light bands on the sides of the cranial region could be influenced by the action at the embryonic level of altered protein turnover mechanisms mediated by the ubiquitin–proteasome–autophagolysosomal system, associated with migration phenomena of the melanosomes’ progenitor cells from the neural tube to the cranial region, giving rise to the typical “*facciuto*” pattern. A similar mechanism involving protein turnover via the syntaxin-17 gene (*STX-17*), known to play a key role in autophagy [80], is described in greying horse breeds [81]. In addition, hair-graying phenotypes have been also observed in humans, such as in (i) Chediak–Higashi syndrome and its analog in several animal species, (ii) Griscelli syndrome, and (iii) Hermansky–Pudlak syndrome and its analog in mice. In all the above-mentioned pathological conditions, the diagnostic hallmarks are represented by defects in the biogenesis or transport of melanosomes, with the latter being specialized members of the lysosomal lineage of organelles [82]. Furthermore, the recurrent presence, in our multi-breed dataset, of genes involved in the cranial development (*AHCY* and *ITCH*) and in the above-described protein turnover mechanisms (*DYNLRB1, MAP1LC3A, PIGU, LOC102177263*, and *DTX4*) leads us to the hypothesis that (i) a possible interplay between these two pathways could be implicated in the “*facciuto*” phenotype in goats and that (ii) an evolutionary conserved mechanism unifying these processes across different Italian breeds may exist.

Overall, the presence of the *ASIP* gene in one of the two supported regions in this study highlights the possible association of this locus with the “*facciuto*” phenotype, a hint further supported by the studies by Guo et al. (2022) [36] and Signer-Hasler et al. (2022) [37] in Western European goat breeds displaying the so-called “Swiss marking” phenotype. To the best of our knowledge, the goat breeds exhibiting the “*facciuto*” phenotype in our study do not genetically directly originate from the Swiss or French breeds considered in [36,37]. However, the molecular mechanism underlying this phenotype may be conserved across breeds within the caprine species, considering the conservation of the *ASIP* gene mechanisms also across different species [3]. Overall, the lack of information about the possible genotypes of the considered “*facciuto*” breeds at the CNVs identified in “Swiss marking” breeds represents a limitation of this work that may be addressed in future studies.

## 4. Conclusions

This study highlighted *ASIP* as a possible candidate for pigmentation patterning in Italian “*facciuto*” goats, thus confirming the previous evidence about its association with the coat color patterning process. In addition, other pathways, such as those suggested by genes involved in cranial development and protein turnover, may concurrently contribute to the determinism of the considered “*facciuto*” phenotype in Italian goats. Furthermore, the implication of genes related to protein turnover in other livestock species, as well as in humans, suggests evolutionary conserved mechanisms underlying depigmentation patterns in mammals, highlighting the importance of adopting cross-species integrative approaches. Overall, this study contributes to generating knowledge about genetic loci associated with the “*facciuto*” phenotype in six Italian local goat breeds, extending our understanding on a trait that represents a hallmark characteristic for most of them, while being still not fixed in others. As such, the information generated in this study opens the way for further investigations to refine and validate, in independent larger datasets, the observed selection signals for assisting breeding decisions toward breed standardization. Notably, studies based on long-read sequencing or high-density SNP data are needed to gain a deeper insight into the complexity at the *ASIP* locus, for which it was not possible, in this study, to investigate CNVs potentially responsible to the phenotype under investigation, and other candidate loci in the goat genome.

## Figures and Tables

**Table 1:** Outline of the adopted genotype datasets and experimental design.

Pairwise Tests	“*facciuto*” Breeds	Breed Code	No. *	“*non-facciuto*” Breeds	Breed Code	No. *
1	Capestrina	CAP	22	Saanen	SAA	43
2	Capestrina	CAP	22	Rossa Mediterranea	RME	46
3	Capestrina	CAP	22	Malagueña	MAL	42
4	Facciuta Lucana	LUC	16	Saanen	SAA	43
5	Facciuta Lucana	LUC	16	Rossa Mediterranea	RME	46
6	Facciuta Lucana	LUC	16	Malagueña	MAL	42
7	Facciuta della Valnerina	VAL	24	Saanen	SAA	43
8	Facciuta della Valnerina	VAL	24	Rossa Mediterranea	RME	46
9	Facciuta della Valnerina	VAL	24	Malagueña	MAL	42
10	Roccaverano	ROC	23	Saanen	SAA	43
11	Roccaverano	ROC	23	Rossa Mediterranea	RME	46
12	Roccaverano	ROC	23	Malagueña	MAL	42
13	Teramana	TER	20	Saanen	SAA	43
14	Teramana	TER	20	Rossa Mediterranea	RME	46
15	Teramana	TER	20	Malagueña	MAL	42
16	Valfortorina	VLF	27	Saanen	SAA	43
17	Valfortorina	VLF	27	Rossa Mediterranea	RME	46
18	Valfortorina	VLF	27	Malagueña	MAL	42

* Number of animals with the “*facciuto*” or “*non-facciuto*” phenotype in the pairwise test.

**Table 2:** Identification of loci potentially under the selection.

Chromosome	SNP ID	Position (bp)	Genes in the ±200 kb Range
13	rs268286785	63,340,917	*LOC102190531; ASIP; **AHCY**; **ITCH**; DYNLRB1; MAP1LC3A; PIGU*
15	rs268264603	942,715	*TRNAR-UCU, TRNAV-UAC, LOC102169614, LOC102169319, LOC102175784, LOC102175501, LOC102175229, LOC102174956, LOC102174680, LOC102174413, LOC102169036, LOC102174141, LOC102168753, **LOC102168475**, **LOC102173289**, LOC102168904, LOC102177738, LOC102191259, LOC102190979, LOC108637643, LOC102177263, DTX4, LOC108637644*

In bold, the genes neighboring the two SNP loci potentially under selection.

## Data Availability

Part of the data used in this study are publicly available (https://data.mendeley.com/datasets/hnd59x6gmg/1, accessed on 11 February 2025). The remaining data are available through contacting the AdaptMap Consortium and the corresponding author.

## Supplementary Materials

The following supporting information can be downloaded at: https://www.mdpi.com/article/10.3390/genes16040390/s1, Supplementary Table S1: Details of the six Italian populations displayed the “*facciuto*” phenotype; Supplementary Table S2: Details of the three breeds with “*non-facciuto*” phenotype; Supplementary Table S3: Significant loci detected through a multi-cohort FST-outlier approach (q-value < 0.05) for each of the 18 pairwise comparisons among the six “*facciuto*” populations and the three “*non-facciuto*” breeds considered in this study; Supplementary Table S4: Significant loci with q-values < 0.05 in ≥50% of the six pairwise comparisons for each scenario involving the six “*facciuto*” populations contrasted with any of the three “*non-facciuto*” breeds. In bold are the loci that were observed as putative under differential selection in at least two out of the three “*non-facciuto*” scenarios; Supplementary Table S5: Results of BayeScan analysis contrasting the six “*facciuto*” breeds, merged into a single population, with the three “*non-facciuto*” comparison breeds; Supplementary Table S6: Minor allele frequencies for the two SNP loci within the nine considered breeds; Supplementary Table S7: Literature evidence supporting the involvement in melanogenesis and/or in other coat color-related mechanisms of the genes annotated on the regions putatively under selection on chromosomes 13 and 15.
