# Supplementary material for: Selection Signatures in Italian Goat Populations Sharing the “facciuto” Phenotype
Source: Genes (Basel). 2025 Mar 28;16(4):390. doi: 10.3390/genes16040390 (PMC12027434; doi:10.3390/genes16040390)
Supplement: Supplementary file 1 [file genes-16-00390-s001.zip › Supplementary_S2.pdf]

Supplementary Table S2: Details of the three breeds with "*non-facciuto*" phenotype.

Name	Phenotype	Origin and peculiarities
Red Mediterranean	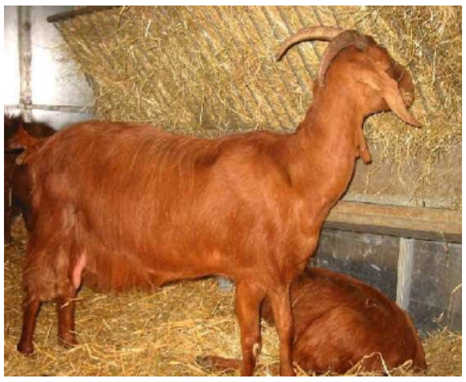	It is a breed of goat originated in the Middle East. Nowadays, it is bred in southern Italy (Sicily, Basilicata and Calabria regions) to produce milk to be used in cheese making. It is classified as “endangered”.
Saanen	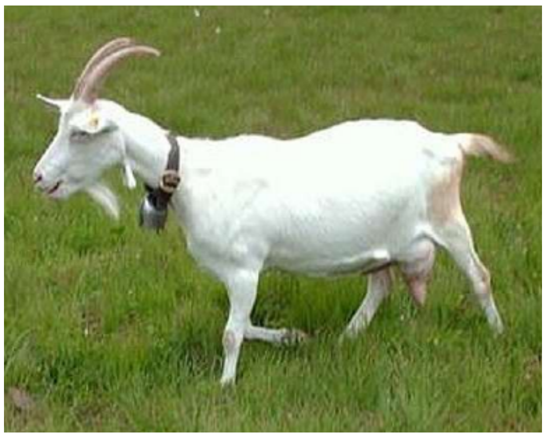	It is an international breed original from Switzerland and nowadays the most widespread goat breed throughout Europe. In Italy, it is most widespread in the Alpine regions, but it is also bred in the rest of the Italian Peninsula. It is a highly productive dairy goat and its milk is used to obtain fresh and aged cheeses, yoghurt and ricotta. It is classified as “not at risk”.
Malagueña	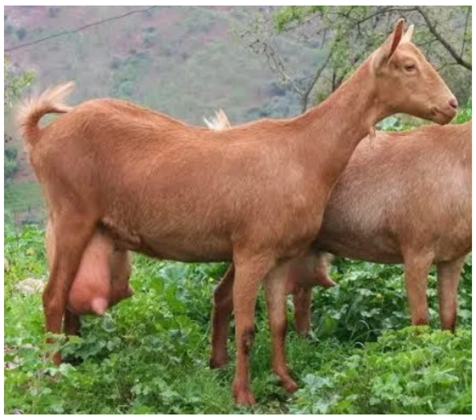	It is most concentrated in Andalusia (especially in the province of Malaga), although it is also found in Extremadura and Castilla y Leon. It is classified as “not at risk”.
