# Supplementary material for: Selection Signatures in Italian Goat Populations Sharing the “facciuto” Phenotype
Source: Genes (Basel). 2025 Mar 28;16(4):390. doi: 10.3390/genes16040390 (PMC12027434; doi:10.3390/genes16040390)
Supplement: Supplementary file 1 [file genes-16-00390-s001.zip › Supplementary_S1.pdf]

Supplementary Table S1: Details of the six Italian breeds displaying the *"facciuto"* phenotype.

**Breed name**	**Phenotype**	**Origin and peculiarities**	**Recognition status**[1]	**Conservation status**[2]
Facciuta Lucana	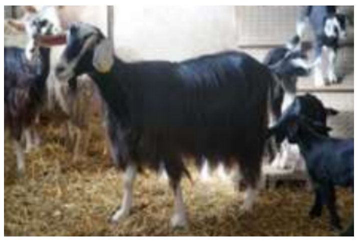	Local goat population from Basilicata region (Southern Italy). It is currently raised both for milk production, from which high-quality cheeses are obtained (i.e. Canestrato di Moliterno), and for meat production given the high twinning.	In August 2024, the "Facciuta Lucana" has been included in the "Disciplinare del libro genealogico e del registro anagrafico della specie caprina".	Not available
Facciuta della Valnerina	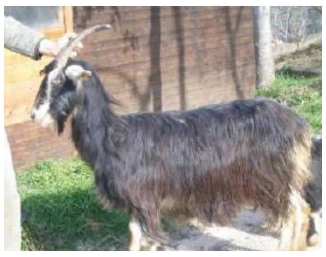	Widespread in the Apennine area between Marche and Umbria regions, being particularly suited to breeding in the high mountains.	Recently recognized as a breed in the latest goats Central Technical Commission of AssoNaPa, the registration process in the genealogical book is currently ongoing.	Not available
Valfortorina	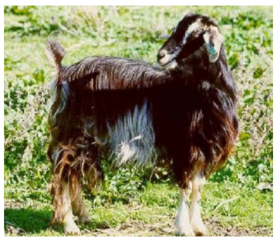	It is a goat population raised in the province of Benevento (Campania region, Southern Italy). Its conservation status is listed as "critical".	Included in the "Anagrafe nazionale della biodiversità di interesse agricolo e alimentare", published by the MASAF through the D.M. n. 156997 of 15 March 2023.	Critical
Teramana	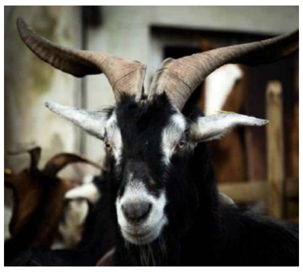	It is raised in the Abruzzo region (Central Italy) and takes its name from the province of Teramo, where these animals have their greatest concentration. Its conservation status is listed as "critical".	Included in the "Anagrafe nazionale della biodiversità di interesse agricolo e alimentare", published by the MASAF through the D.M. n. 156997 of 15 March 2023. Registered in the AssoNaPa Herd Book of goat breeds with a genetic conservation program.	Critical
Capestrina	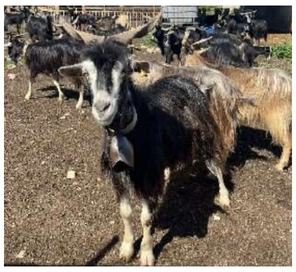	It is an indigenous breed of domestic goat from the mountain area in the Southern part of Lazio region (Central Italy). It is classified as "endangered".	Included in the "Anagrafe nazionale della biodiversità di interesse agricolo e alimentare", published by the MASAF through the D.M. n. 156997 of 15 March 2023. Registered in the AssoNaPa Herd Book of goat breeds with a genetic conservation program.	Endangered
Roccaverano	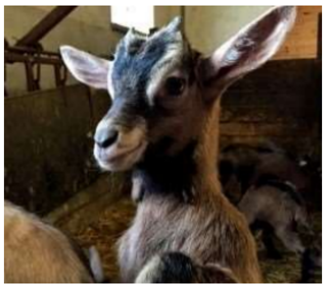	Native to the Piemonte region (Northern Italy), it is a breed with a prominent aptitude for milk production, from which Robiola di Roccaverano, a cheese with D.O.P. since 1996 (EC Reg. n.1263), is produced. It is classified as "endangered".	Included in the "Anagrafe nazionale della biodiversità di interesse agricolo e alimentare", published by the MASAF through the D.M. n. 156997 of 15 March 2023. Registered in the AssoNaPa Herd Book of goat breeds with a genetic conservation program.	Endangered

[1]Available information about the recognition status of the breed; [2]detailed local risk status according to the DAD-IS database by the FAO.
